# |tPRiors |: a tool for prior elicitation and obtaining posterior distributions of true disease prevalence

**DOI:** 10.1186/s12874-022-01557-1

**Published:** 2022-04-03

**Authors:** Konstantinos Pateras, Polychronis Kostoulas

**Affiliations:** Laboratory of Epidemiology & Artificial Intelligence, Faculty of Public and One Health, University of Thessaly, Karditsa, Greece

**Keywords:** Prevalence estimation, Prior elicitation, Bayesian, Pooled samples, Statistical modelling, JAGS, Shiny

## Abstract

**Background:**

Tests have false positive or false negative results, which, if not properly accounted for, may provide misleading apparent prevalence estimates based on the observed rate of positive tests and not the true disease prevalence estimates. Methods to estimate the true prevalence of disease, adjusting for the sensitivity and the specificity of the diagnostic tests are available and can be applied, though, such procedures can be cumbersome to researchers with or without a solid statistical background. This manuscript introduces a web-based application that integrates statistical methods for Bayesian inference of true disease prevalence based on prior elicitation for the accuracy of the diagnostic tests. This tool allows practitioners to simultaneously analyse and visualize results while using interactive sliders and output prior/posterior plots.

**Methods - implementation:**

Three methods for prevalence prior elicitation and four core families of Bayesian methods have been combined and incorporated in this web tool. |*tPRiors*| user interface has been developed with R and Shiny and may be freely accessed on-line.

**Results:**

|*tPRiors*| allows researchers to use preloaded data or upload their own datasets and perform analysis on either single or multiple population groups clusters, allowing, if needed, for excess zero prevalence. The final report is exported in raw parts either as.rdata or.png files and can be further analysed. We utilize a real multiple-population and a toy single-population dataset to demonstrate the robustness and capabilities of |*tPRiors*|.

**Conclusions:**

We expect |*tPRiors*| to be helpful for researchers interested in true disease prevalence estimation and who are keen on accounting for prior information. |*tPRiors*| acts both as a statistical tool and a simplified step-by-step statistical framework that facilitates the use of complex Bayesian methods. The application of |*tPRiors*| is expected to aid standardization of practices in the field of Bayesian modelling on subject and multiple group-based true prevalence estimation.

## Background

Apparent prevalence represents the observed rate of positive test results. Tests can give false positive and false negative results, thus apparent prevalence estimates are biased estimates of the true disease prevalence, while the extend of this bias depends on the overall misclassification rate of testing. Methods are available and can be applied to estimate the true prevalence of disease and, thus, adjust for the imperfect sensitivity and specificity of the diagnostic tests, either within a frequentist or a Bayesian framework [1, 2]. The primary advantage of the latter framework lies in its ability to incorporate in the estimation process existing and relevant knowledge for the parameters of interest in the form of priors [3]. Nonetheless, the application of such approaches can be cumbersome for researchers with or without a statistical background, which results in limited application of existing Bayesian methods for calculation of true prevalence by non-experts. A statistical program that encompasses most of these prevalence models would boost the application of such methods and aid practitioners to perform basic and advanced Bayesian prevalence estimation.

In this manuscript we present Bayesian models for the estimation of apparent and true disease prevalence for a single and multiple populations (“Implementation section). Then, we introduce a new free web-based application, |*tPRiors*|, that facilitates the use of Bayesian prevalence methods by non-experts (“The |*tPRiors*| web-based application” section), which has been developed with R and Shiny [4]. Finally, in “Results” section, we illustrate the use of |*tPRiors*| using a single toy population example [5], and a real multiple population example that is based on a systematic review and meta-analysis on the prevalence of Dementia in Europe [6]. The manuscript ends with a Discussion.

## Implementation

### Methods for prevalence estimation

#### Apparent prevalence estimation

Let us assume that in a single population of size *N* (i.e. nation, group of patients, animal herd or any cluster) *y* subjects test positive out of the *n* randomly sampled population subjects. Then *y* follows, approximately, a binomial distribution:

1 $$ \begin{aligned} y|\pi_{a}& \sim Binomial(n,\pi_{a})\\ \pi_{a} & \sim Beta(\alpha_{\pi_{a}},\beta_{\pi_{a}}) \end{aligned}  $$

where *π*_*a*_ is the apparent disease prevalence and $\alpha _{\pi _{a}},\beta _{\pi _{a}}$ are the parameters of the Beta prior distribution for *π*_*a*_. The posterior distribution of the above model can be analytically calculated from $Beta(\alpha _{\pi _{a}}+y,\beta _{\pi _{a}}-y+n)$ or through the use of Bayesian statistical programs (WinBugs, Jags, Stan) [7–9]. In the absence of historical information, parameters $\alpha _{\pi _{a}},\beta _{\pi _{a}}$ can be set close or equal to 1, which is similar to assuming a Uniform(0,1) prior on *π*_*a*_. Relevant existing knowledge for *π*_*a*_ can be incorporated by specifying values for $\alpha _{\pi _{a}},\ \beta _{\pi _{a}}$ that leads to a prior *Beta* distribution that captures such knowledge. |*tPriors*| can be used to specify these values (see “The |*tPRiors*| web-based application” section).

#### True prevalence estimation

The relationship between apparent (*π*_*a*_) and true disease prevalence (*π*_*t*_) is known and given by *π*_*a*_=*π*_*t*_*S*_*e*_+(1−*π*_*t*_)(1−*S*_*p*_) [10], where *S*_*e*_,*S*_*p*_ denote the sensitivity and specificity of the diagnostic test. Hence, replacing *π*_*a*_ with the above in Eq. 1,

2 $$ \begin{aligned} y|\pi_{t},S_{e},S_{p} & \sim Binomial(n,\pi_{t}\cdot S_{e}+(1-\pi_{t})\cdot(1-S_{p})) \\ \pi_{t} & \sim Beta(\alpha_{\pi_{t}},\beta_{\pi_{t}}) \\ Se & \sim Beta(\alpha_{S_{e}},\beta_{S_{e}}),\ Sp\sim Beta(\alpha_{S_{p}},\beta_{S_{p}}) \\ \end{aligned}  $$

where $\alpha _{\pi _{t}},\beta _{\pi _{t}},\alpha _{S_{e}},\beta _{S_{e}},\alpha _{S_{p}},\beta _{S_{p}}$ are the parameters of the Beta prior distributions for *p*_*t*_,*S*_*e*_ and *S*_*p*_, respectively [2].

#### Multiple populations

Let us suppose that *n*_*i*_, *i*=1,2,...,*k* subjects are sampled randomly from multiple clusters (*k*). When multiple clusters are available, commonly a two-stage cluster-design is implemented. In the first stage *k* clusters are selected randomly within a region and then *n*_*i*_ subjects are sampled randomly from each cluster (*i*). In the end, *y*_*i*_ positive tests will be collected from each cluster. As in the single cluster case above, the number of tests, are assumed to follow a binomial distribution [1],

3 $$\begin{array}{*{20}l} {}y_{i}|\pi_{ti},S_{e},S_{p} & \sim Binomial(n_{i},\pi_{ti}S_{e}+(1-\pi_{ti})(1-S_{p})) \end{array} $$

4 $$\begin{array}{*{20}l} \pi_{ti}|\mu,\psi & \sim Beta(\mu\psi,\psi(1-\mu))\notag \\ S_{e} & \sim Beta(\alpha_{S_{e}},\beta_{S_{e}}),\ S_{p}\sim Beta(\alpha_{S_{p}},\beta_{S_{p}})\notag \\ \mu & \sim Beta(\alpha_{\mu},\beta_{\mu}),\ \psi \sim Gamma(\alpha_{\psi},\beta_{\psi}) \notag \\ \end{array} $$

*S*_*e*_ and *S*_*p*_ are modelled through beta prior distributions and are assumed invariant between clusters. The sampled group prevalences are considered exchangeable as *π*_*ti*_|*μ*,*ψ*∼*Beta*(*μψ*,*ψ*(1−*μ*)), where *μ* denotes the average population prevalence and *ψ* is associated with the heterogeneity (between-group variance) of cluster prevalences around their mean. *μ*,*ψ* are modelled through a Beta and Gamma prior distributions respectively. Large values of *ψ* correspond to lower between-study variability (heterogeneity). On all hyper-parameters (i.e. *α*_*μ*_,*β*_*μ*_,*α*_*ψ*_,*β*_*ψ*_) a hyper-prior distribution can be assumed, also, all hyper parameters can be elicited based on expert opinion. A logical strategy, though, would be to reduce the number of unknown parameters, given that the data collection procedure relies solely on a basic binomial count [2].

#### Allowing for zero true prevalence

All models above assume a flexible beta prior distribution on *π*_*t*_. This implies that *π*_*t*_≠0, an assumption that may not be realistic on low prevalence populations or on populations with specific free from disease clusters. To account for the probability of zero disease prevalence, a mixture distribution can be applied such that $\phantom {\dot {i}\!}\pi _{t}\sim Beta(\alpha _{\pi _{t}},\beta _{\pi _{t}})$ for a single population or *π*_*ti*_|*μ*,*ψ*∼*Beta*(*μψ*,*ψ*(1−*μ*)) for multiple populations with probability *w*, and *π*=0 with probability 1−*w*, where *w* is the probability that the group is actually infected [2].

$$\pi^{*} =\left\{ \begin{array}{ll} \pi_{t} \;\;\; with \;\;\; probability \;\;\; w \\ 0 \;\;\;\; with \;\;\; probability \;\;\; 1-w \end{array}\right. $$

In a similar sense, a beta prior distribution can be placed on *w*, with *α*_*π*_,*β*_*π*_=1 denoting prior ignorance. It should be noted that omitting to place a mixture distribution on *π*_*a*_ may result in biases and possibly narrower credible intervals. Following the multiple population modelling, *w* can be further modelled similarly to *π*_*ti*_ to allow for zero prevalence in the whole region [11].

The multinomial model allows for estimation of (a) the group-level prevalence, (b) the prevalence distribution for groups, (c) the mean of the prevalence distribution, (d) the probability that the whole (sub) population is free of disease (at a pre-specified level) and finally (e) the predicted probability that a group is free of disease (again at a pre-specified level).

### The |*tPRiors*| web-based application

|*tPRiors*| is an open-access web-based application with a graphical, user-friendly interface which aids the estimation of true disease prevalence from single and multiple population data using the models presented in “Implementation” section. Non-expert users on Bayesian analysis and relevant software, such as R/JAGS, can easily elicit priors, perform Bayesian statistical analysis, visualize results and download the corresponding reports.

One of the mechanisms of the |*tPRiors*| application is the ”PriorGen” R package (Kostoulas, 2018), which powers the prior elicitation by translating prior beliefs into usable prior information in the form of hyper-parameters for the Beta (and Gamma) distributions. This package can be utilized for the generation of priors on the true prevalence of disease, the sensitivity and the specificity of diagnostic tests and the probability of zero true prevalence.

|*tPRiors*| is powered by Shiny, thus, it has be deployed on shinyapps.io that allows for easy user access without the need for installation of R or JAGS. The web application can be accessed from any device that has internet connection and a web browser, even though more optimal use is warranted via a computer or a laptop. |*tPRiors*| is currently freely accessible to download and use in R/Rstudio via https://github.com/kpatera/tPRiors or it can be directly accessed via https://publicandonehealth.shinyapps.io/tPRiors/. The application can be accessed through different operating systems and internet browsers, though, we recommend the use of an updated Google Chrome or Firefox browser.

### |*tPRiors*| step by step walkthrough

**Fig. 1:**
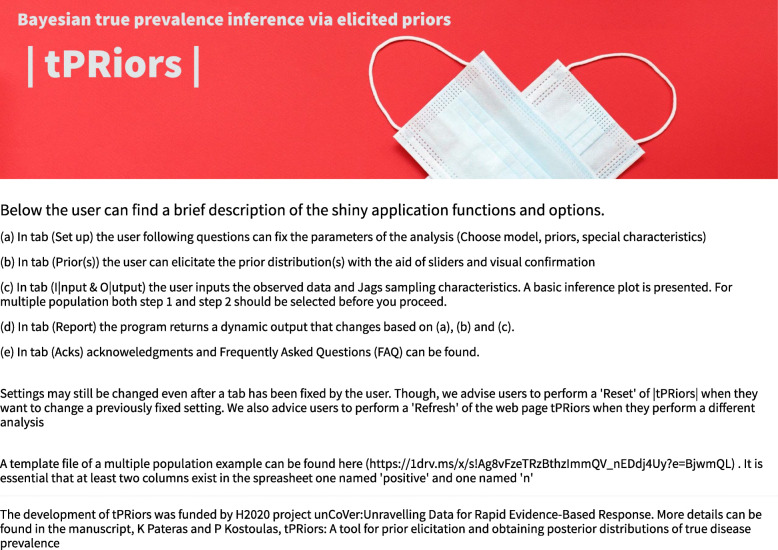
Initial screen and menus of the |*tPRiors*| web-based application. This screen-shot showcases the application and it is not meant for direct reading. For a dynamic web-page one should visit the link https://publicandonehealth.shinyapps.io/tPRiors/

The initial ”Start” page and the other menus of |*tPRiors*| are presented in Fig. 1. |*tPRiors*| consists of six individual pages/tabs: 1. Start, 2. Set-up, 3. Prior(s), 4. I |nput & O |utput, 5. Report and 6. Acks. Three of these pages, “Set up”, “Prior(s)” and " I |nput & O |utput “, contain the core mechanisms. On each page the user has to press the ”Fix/Set" button, after choosing the preferred options and before clicking on the next |*tPRiors*| page. Frequently asked questions (FAQ) can be found in the ’Acks’ page. A description for each page follows.

#### Start

The first page provides a general overview of |*tPriors*| and a brief description on how the user should proceed.

#### Set up

In this page the users select the modelling framework that best captures the structure of their data/population. The users first specify whether they will analyse data from one or multiple populations/clusters. Then, they define whether they wish to estimate true or apparent prevalence. Subsequently, they need to decide whether they wish to allow for the possibility of zero prevalence for some of the populations/clusters. Finally, they indicate whether there is a priori information for the prevalence (apparent or true) that they wish to use and then they decide which measure of central tendency (mean, median or mode) or dispersion (percentiles) they will employ for this prior specification on prevalence. The multiple population models option currently supports one prior specification measure (the mean).

#### Prior(s)

In this page the users should specify the available information that will be used for generation of the prior distribution for apparent or true prevalence. If, in the previous page, they have declared that the they are estimating true prevalence then two additional sub-pages become available for the sensitivity and specificity of the diagnostic test. Clicking on the ‘Help’ button will provide an example on how to elicit a prior based on the available questions. We urge users of |*tPRiors*| to first answer all questions, input values on all available parameters, and only then press the ’Set priors’ button, in this way small subjectivity would be inserted in the elicitation proccess. Prior specification is a guided-through process, though, further guidance can be found when the ’Help’ button is clicked or in the FAQ section of the ’Acks’ page. Furthermore, additional information can be found at the PriorGen manual [12].

#### I & O (I|nput & O|utput)

Based on their selection in the “Prior(s)” page, in this page users should input and model either single or multiple population data. For single population data, users should specify via a number of sliders, the sample size, number of events and Markov Chain Monte Carlo (MCMC) - related options. For multiple-population data, users should upload a dataset (.csv/.xls). Each row of the data table should correspond to a different site/study and the dataset should contain at least 2 columns. One column named “*positive*” that contains the observed number of events per site/population and one column named “*n*” that contains the sample sizes per site/population. When all parameters are set the user should then press the ’Fix model’ button and only then click on the “Report” page. Caution! Both Step 1. Data and Step 2. Output tabs have to be clicked to fully run the multiple-population models.

#### Report

**Fig. 2:**
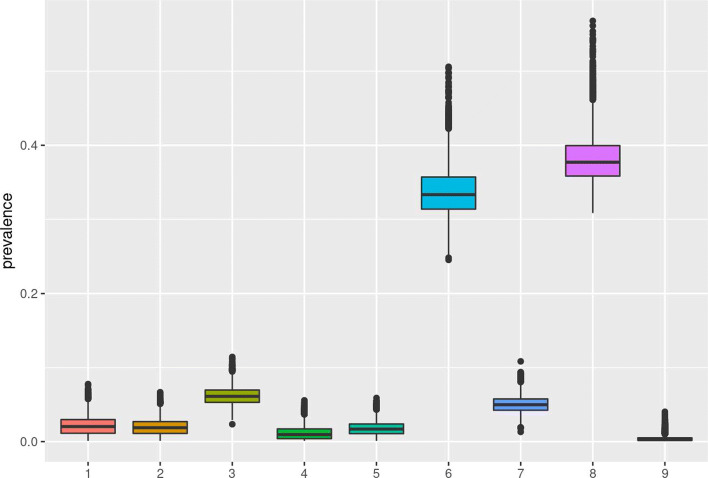
Box plots with estimates for each one of the nine studies presented in Table 2 - |*tPRiors*| output

After navigating through the above pages, users find a dynamic report in the fifth page. This report contains all previous selected options, information on the selected prior distributions as well as posterior prevalence inferences and MCMC diagnostics to check convergence. All figures can be directly downloaded. In the case of multiple populations, box plots are provided for each study in a dynamic graph (Fig. 2), together with information that aims to aid researchers in planning an upcoming prevalence study.

Near the end of the report a series of diagnostic plots are presented. More specifically, barplots and density plots of the main parameters are available at the top of the section, alongside traceplots and running mean plots, followed by complete versus partial chain plots and autocorrelation plots of the main parameters. When the MCMC analysis runs into issues users will identify non-common patterns in these graphs. Mostly diagnostic plots point towards convergence issues. The simple density plots can show abnormalities in the posterior densities, if any density look different than expected the user should check the data and prior inputs. The multiple chain or partial chain density plots should contain densities of different chains that mostly coincide, otherwise posterior Bayesian convergence is impacted by each MCMC run and the results may not be trusted. MCMC traceplots should depict multiple chains that often cross in order to validate convergence while, the ergodic mean plots and the correlation plots should move very quickly towards the mean and zero respectively, otherwise the user should increase the thinning interval and/or the number of MCMC samples. We refer interested readers and practitioners to the CODA and GSS R packages and we suggest that, when in doubt, they should always consult with a statistician.

Finally, |*tPRiors*| provides further access to the analysis by making available three files at the bottom of the ”Report” page and analytical details on how to re-analyse them. These files are available under the 1) Input, 2) Model and 3) Output tabs. Interested users have the option to utilize Rstudio or R to work with the specific rData file of interest. By utilizing these files, i.e. study-level plots can be re-produced to identify and diagnose further methodological issues or change the appearance and labels of the figures. Detailed steps on how to reproduce diagnostic plots can be found at the end of the “report” page, right before the download data tabs. Briefly, the MCMC samples can be downloaded from the “Report” page via the ’Output’ tab. Within the downloaded object, multiple MCMC chains are stored for each parameter of interest. Following the detailed steps on the “Report” page, practitioners can repetition (mentioned right above) attempt further analyses.

#### Acks

In the final page/tab an overview of the dependencies on R packages can be found. |*tPRiors*| web application was built using a number of R packages such as: shiny, shinydashboard, rmarkdown. R2jags, rootSolve, reshape, ggplot2, dplyr, DT and plotly among others. The different colours represent the four main categories of use., i.e. the orange colour denotes main packages, the green colour denotes shiny-based packages, blue and variations denote packages that aid plotting/reporting and the red colour denotes packages for data manipulation.

## Results

The following section presents two, a multiple and a single population, examples of true prevalence estimation while taking into account the sensitivity and specificity of the applied (imperfect) diagnostic test.

### Multiple population illustrative example

The multinomial approach estimates true prevalences that can directly be compared among the multiple study sites (i.e. studies, regions, cities, groups) as they are adjusted for imperfect diagnostic testing.

We present the systematic review on prevalence from Bacigalupo et al. [6]. The authors conducted a systematic review and meta-analysis on dementia and Alzheimer’s disease. They selected studies that diagnosed patients according to the DSM (Diagnostic and Statistical Manual of Mental Disorders) IV criteria but also studies that had a high quality score according to Alzheimer’s Disease International study quality score criteria [13]. The meta-analysis included 9 studies conducted in Europe between 1993 and 2018 with patients aged >65 years. The total number of participants were 18263, out of which 2137 were diagnosed with dementia based on DSM IV criteria. The meta-analysed pooled prevalence rate estimate was reported as equal to 12.4% (95% CI 7.6%–17.2%). The raw data are summarized in Table 1.

**Table 1:** Studies included in the systematic review of Bacigalupo et al. 2018 alongside their apparent prevalence

ID	Study	Country	Year(s)	Positive (*y*_*i*_)	N (*n*_*i*_)	App. Prev (*π*_*α*_)	95% CIs
1	Ravaglia et al.	Italy	1999	60	961	0.062	(0.047,0.078)
2	Tognoni et al.	Italy	2000	100	1662	0.060	(0.049,0.072)
3	Gascon-Bayarri et al.	Spain	2002	165	1754	0.094	(0.080,0.108)
4	Fish et al.	UK	2003	88	1754	0.053	(0.042,0.064)
5	Bermejo-Pareja et al.	Spain	1994-5, 1997-8	306	5278	0.058	(0.052,0.064)
6	Mathillas et al.	Sweden	2000-2, 2005-7	287	895	0.321	(0.290,0.351)
7	Tola-Arribas et al.	Spain	2009-10	184	2170	0.085	(0.073,0.097)
8	Lucca et al.	Italy	2009-10	894	2501	0.357	(0.339,0.376)
9	Perquin et al.	Luxembourg	2008	53	1377	0.038	(0.028,0.049)

A number of subjects have been tested (n_i_) per study/country, out of which (y_i_) subjects showed positive test results. The test procedure was based on DSM IV criteria for all participants. In comparison with previously used diagnostic procedures, the DSM IV criteria result in higher specificity. On average, based on historical data and previous studies, sensitivity may be set equal to 95% and specificity can be set equal to 88% [14–16], though if co-morbidities or mild dementia are present, specificity may drop even further [17].

On |*tPRiors*|, the user selects to model 1) true prevalence, 2) multiple populations, 3) non-zero true prevalence, 4) informative priors^1^ and then clicks fix. Consequently, in the “Prior(s)” page and given the above information, the researcher specify priors on the disease prevalence and the test specificity and sensitivity. Via the use of simple sliders in the tab “Priors”, a low-informative prior is elicited for the true prevalence, as a *π*_*t*_∼*Beta*(0.14,0.55). The |*tPRiors*| shiny application input for this prevalence prior would be: *π*_*t*_(0.2, 0.99, 0.3, 0.986). This prior has a median equal to 1.6% and IQR (Interquartile range) equal to (0.06%, 39.03%) denoting our limited knowledge on the true prevalence for dementia in Europe. Similarly, for the specificity parameter *S*_*p*_ ∼*Beta*(25,3.5) is elicited, while for the sensitivity parameter *S*_*e*_ ∼*Beta*(25.6,1.4) is elicited based on the user inputs on the |*tPRiors*| “Prior(s)” page. The |*tPRiors*| shiny application input for the sensitivity and specificity priors would be: *S*_*e*_(0.88, 0.80, 0.90) - *S*_*p*_(0.95, 0.85, 0.97)]. The sensitivity prior implies a prior median value of 0.88 (IQR: 0.85, 0.92)and the specificity prior implies a prior median value of 0.95 (IQR: 0.93, 0.98).

**Analysis and report**

Regarding our multiple-population motivating example, after running the analysis with the defined priors, settings and uploaded datasets, the following statistics are reported by the |*tPRiors*| application. The reported probability that the posterior true prevalence would be higher than 0.05 was equal to 1. The prevalence posterior mean equals to 18.5% which is larger than the original pooled estimate of the Bacigalupo et al. review and may imply less modelled heterogeneity and thus larger weights on Mathillas *et al* (6) and Lucca *et al* (9) studies. In comparison with the simple apparent prevalence estimates, the lower studies’ prevalence shrunk towards zero, while study 6 and 9 showed an increase in prevalence when accounting for imperfect testing, a behaviour that is partially explained by the test’s low specificity. In this example, we did not model the probability of zero prevalence as we assumed that the dementia prevalence would not be zero in the age groups studied.

### Single population illustrative example

In this section we re-analyse a single population toy example to reproduce a previously published analysis from Spreybroek et al. [5]. In their example, for simplicity the authors have assumed a sample size of 500 with two assumed apparent prevalences, one equal to 24% that corresponds to a number of positives equal to 120 and one equal to 5% that corresponds to a number of positives equal to 25. Spreybroek et al. considered two Bayesian models. They have assumed either 1) a fixed value for the sensitivity (*S*_*e*_=0.80) and specificity parameter *S*_*p*_=0.90), or 2) a uniform distribution for the two parameters *S*_*e*_∼*Unif*(0.7,0.9) and *S*_*p*_∼*Unif*(0.85,0.95). We partially adopt their assumptions by setting Beta distributions on prior parameters with mean test sensitivity equal to 80% and mean test specificity equal to 90%, while avoiding the use of fixed values (Table 2). As prior probability for the true prevalence we elicit a a) *π*_*t*_∼*Beta*(1,1), denoting Setup A and a b) *π*_*t*_∼*Beta*(1.3,1.3), denoting Setup B. Both setups express our belief that, before observing the data all values are likely or almost equally likely possible for the true prevalence. In setup A we assume that the mean value of the true prevalence equals 0.5 and it is uniformly distributed around this value. The *π*_*t*_∼*Beta*(1.3,1.3), prior distribution on the true prevalence of Setup B has a mean value of 0.5 (95% CrI 0.28,0.72). The |*tPRiors*| application shiny input for setup A would be: [ *π*_*t*_(0.5, 0.05, 0.95) - *S*_*e*_(0.8, 0.75, 0.97) - *S*_*p*_(0.9, 0.85, 0.97)]. Setup B places slightly smaller weight in prior true prevalence values close to 0/1, a plausible assumption as we expect that the prevalence would neither be very small nor very large. The |*tPRiors*| application shiny input for setup B would be: [ *π*_*t*_(0.5, **0.08**, 0.95) - *S*_*e*_(0.8, 0.75, 0.97) - *S*_*p*_(0.9, 0.85, 0.97)]. For the sensitivity and specificity parameters in all scenarios we assume a mean value of 0.8 and 0.9 with a 97% probability that the mean values are higher than 0.75 and 0.85 respectively. Therefore, both Setup A and Setup B place the same prior on the sensitivity (0.8, CrI: 0.78, 0.82) and the same prior on the specificity (0.9, CrI: 0.88, 0.92). For both setup A and setup B, the user is able to impose a non-zero prevalence prior, if the prior probability of this prior is low, posterior inference would set additional density weight on zero. Let us assume that when non-zero true prevalence is selected, the |*tPRiors*| application shiny input for the non-zero true prevalence alternative could be: [ *w*(0.2, 0.1, 0.95)], meaning that based on the available literature the mean value for non-zero prevalence probability is expected to be equal to 0.2 and we can be 95% sure that it is higher than 0.1.

**Table 2:** True prevalence results (mean and quantile of the 95% credible interval (Q) of the toy example with 24% and 5% apparent prevalence, 500 sample size, 120/25 positive tests with consistent assumptions on sensitivity (80%) and specificity (90%). Speybroek et al. 2012 Model 1 assumes fixed values for *Se* and *Sp*, while Model 2 assumes *Se*∼*Unif*(0.7,0.9) and *Sp*∼*Unif*(0.85,0.95). For the |*tPRiors*| example, *Se*∼*Beta*(195.76,48.94) and *Sp*∼*Beta*(135.86,15.1), while for Setup A: *π*_*t*_∼*Beta*(1,1) and Setup B: *π*_*t*_∼*Beta*(1.3,1.3)

	Speybroek et al. 2012				tPRiors			
	Model 1		Model 2		Setup A.		Setup B.	
Measure	24%	5%	24%	5%	24%	5%	24%	5%
Mean	0.2015	0.0048	0.2008	0.0096	0.2004	0.0103	0.2026	0.0127
2.5% Q	0.1493	0.0001	0.1133	0.0003	0.1266	0.0003	0.1203	0.0008
97.5% Q	0.2577	0.0173	0.2927	0.0336	0.2705	0.0327	0.2764	0.0367

**Analysis and report**

**Fig. 3:**
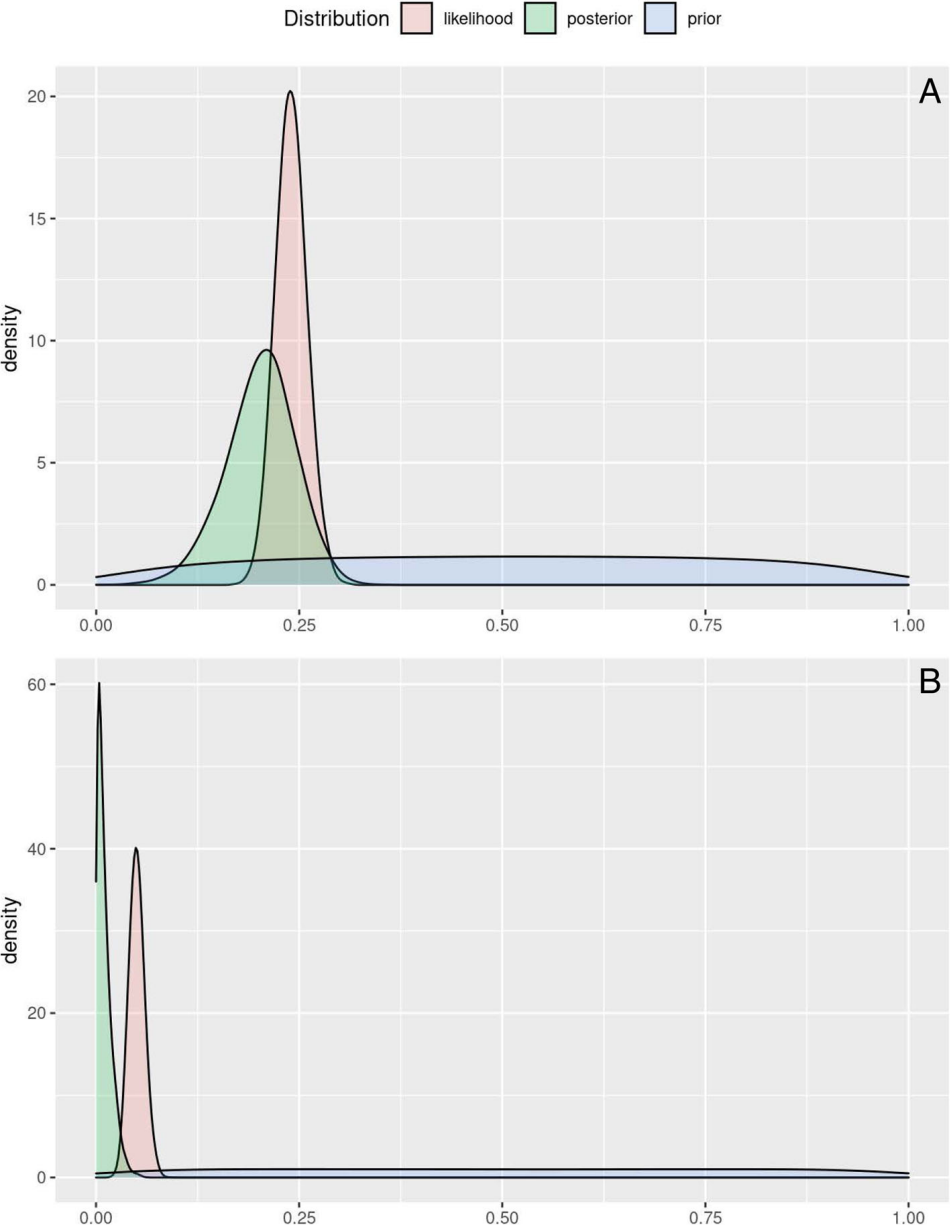
Prior, posterior probability densities alongside the likelihood for Setup B with i. apparent prevalence: 24% or ii. apparent prevalence: 5% of the single population toy example - |*tPRiors*| output

As depicted in Fig. 3(A-B), the posterior distribution of true prevalence points toward a lower prevalence than the apparent prevalence. Table 2 presents the toy example results of the original study [5] and the results produced by the |*tPRiors*| web-based application. The posterior mean of the original manuscript and the web-based application are very similar. Some discrepancies are observed on the width of the 95% credible intervals. More specifically, the |*tPRiors*| Setup A results in intervals (e.g. 24%: CrI (0.13, 0.27)) that lie between the two intervals produced by the original manuscript model 1 (24%: CrI (0.15, 0.25)) and model 2 (24%: CrI (0.11, 0.29)) Table 2]. This result is sensible as our prior assumptions are more relaxed than model 1 but more concentrated than the original model 2 prior distributions in Speybroeck et al. [5]. Finally, regarding the zero-prevalence prior alternative, Setup A, when assuming that the apparent prevalence equals to 5%, the analysis results in posterior mean true prevalence equal to 0.010 (95% CrI: 0.001, 0.032), while when assuming that the apparent prevalence equals to 24%, the analysis results in posterior mean true prevalence equal to 0.1988 (95% CrI: 0.1120, 0.2745). These values should be interpreted with caution, as the resulting posterior distribution is expected to have two peaks as expected and as it can be seen in the |*tPRiors*| ’Report’ page.

## Discussion

In this manuscript, we introduced |*tPRiors*|, a newly implemented web-based application for the calculation of prevalence based on prior information. During recent years such web-based applications have gained considerate popularity, as they are able to streamline and replicate otherwise complex analyses. Several similar applications are available in different areas of medical research and epidemiology as online tools or as statistical packages such as: i) IWA, an application that implemented full hierarchical model for prevalence estimation [11], ii) IPDmada an application that performs individual patient data meta-analysis [18], iii) covid19-tracker, an application that visualizes data from 2019-2020 covid pandemic [19] or iv) shinyCircos for visualizing genomic data [20]. To our knowledge, |*tPRiors*| is the first application that incorporates elicited prior knowledge, transform the latter into prior distributions and calculated posterior inferences for a variety of single and multiple population prevalence models. |*tPRiors*| makes the development and implementation of prevalence Bayesian inferences less cumbersome for researchers with or without strong statistical knowledge. Besides the basic Bayesian inference provided in tPRiors, an important function of such a web-based application is the interactive setting of the prior distributions on the relevant parameters, which can aid researchers towards prior elicitation in a natural and visual way.

Currently, four core models are incorporated in the web-application and “infinite” variations of these basic models can be constructed given input from the user, though, more models may be applied in practice. |*tPRiors*| has been developed as a framework that could encompass more models in the near future, either through a further systematic search or through alternative approaches researched from within our group, in an updated version of this web-application. No comparisons between approaches were performed, hence, interested readers are prompted to read the manuscripts that introduced or critically discussed these models [1,2,5]. To aid researchers, we provide templates/preloaded multiple population datasets, as multiple population data should be uploaded in a specific format. No specific analyses for missing outcome data is currently incorporated, Bayesian inference, however, lends itself to accounting for missing outcome data via proper assumptions. Such considerations were out of scope but can be readily incorporated in the current models. Finally, |*tPRiors*| implements modelling with and without assuming a mixture distributions that allow for zero true infection prevalences. We should note that, especially in populations with low or zero prevalence, even though simpler models may be more intuitive, if mixture models for prevalences are not applied, posterior inferences might be artificially inflated. In practice, we should note that the multiple population models perform more robustly when more than eight clusters are considered.

## Conclusions

The application of |*tPRiors*| is expected to aid standardization of practices in the field of Bayesian modelling on subject and multiple group-based true prevalence estimation. We further believe that |*tPRiors*| will help towards the increase of popularity of the aforementioned methods among practitioners and researchers in the public health sector. Although, | tPRiors | aims to aid researchers to perform Bayesian analysis with ease, we recommend at least one statistician to be part of the research team when such analyses are formally conducted.

**Highlights**

- 1. |*tPRiors*| is a new web-based tool for conducting Bayesian inference of cross-sectional sampling via the use of single or multiple population diagnostic tests results.
- 2. |*tPRiors*| is a free tool, available for all researchers/practitioners and it does not require advanced knowledge or installation of any statistical software (i.e. R, SAS, SPSS).
- 3. |*tPRiors*| is designed to guide the user to the final analysis via the use of simple questions, leading to a printable final report.
- 4. Development of |*tPRiors*| will increase the quality of calculation and reporting of (true) prevalence studies.

## Availability and requirements

**Project name:** |*tPRiors*|

**Project home page:**https://publicandonehealth.shinyapps.io/tPRiors/ and https://github.com/kpatera/tPRiors, publicly released [21]

**Operating system(s):** Platform independent

**Programming language:** R / Shiny

**Other requirements:** Firefox >v96 or another internet browser

**License:** GNU GPL v3.0

**Any restrictions to use by non-academics:** License needed

## Data Availability

All datasets exploited in this manuscript can be found online in the corresponding |*tPRiors*| application specific single or multiple population pages [21].

## Abbreviations

DSM: Diagnostic and statistical manual of mental disorders
IQR: Interquartile range
CrI: Credible interval
FAQ: Frequently asked questions
MCMC: Markov chain Monte Carlo

## Footnotes

1 Do you have single or multiple populations/clusters? Multiple, Do you want to model the true or the apparent prevalence? ? True prevalence, Do you want to account for zero true prevalence? No, Would you specify informative priors? Yes
